# Use of Fecal Sampling for Assessing Probiotic Effects on the Gut Microbiome: A Focused Narrative Review

**DOI:** 10.7759/cureus.115981

**Published:** 2026-09-09

**Authors:** Zvi Weizman

**Affiliations:** 1 Pediatrics, Faculty of Health Sciences, Ben-Gurion University of the Negev, Beer-Sheva, ISR

**Keywords:** feces, gut microbiome, probiotics, sampling, stool

## Abstract

Most clinical studies investigating the effects of probiotics on the gut microbiota rely on fecal samples, which are generally assumed to reflect the gut microbial environment. However, growing evidence indicates that fecal samples do not always accurately represent the gastrointestinal microbiota, particularly the mucosa-adherent microbiome. This focused narrative review examines the limitations of fecal sampling in probiotic research while acknowledging contexts where this approach remains informative. It also discusses methodological strategies that may improve the reliability of microbiome studies, including multi-day sample collection, optimized DNA extraction incorporating bead beating, quantitative microbiome profiling, and ingestible capsule-based sampling. Together, these approaches may provide a more comprehensive assessment of the gut microbiome and improve our understanding of how probiotics influence microbial communities throughout the gastrointestinal tract.

## Introduction and background

The human gastrointestinal tract comprises several distinct ecosystems, each characterized by unique environmental conditions that shape local microbial communities. Despite extensive research on the human microbiome and the effects of probiotics, an important methodological question remains: how accurately do fecal samples represent the gut microbial environment? Stool samples are widely used to assess probiotic effects on the gut microbiota because they are easy and non-invasive to collect. They can capture broad community-level changes, microbial metabolites, and probiotic strain survival during gastrointestinal transit. However, fecal samples may not accurately represent the mucosa-adherent microbiome, particularly when the objective is to evaluate site-specific colonization or mucosal immune interactions. Microbial communities occupying specialized intestinal niches, including crypts, mucus, and mucosal surfaces, may therefore be substantially underrepresented in stool samples [1]. 

The degree of discordance between fecal and mucosal microbiota may also vary with probiotic strain characteristics. Spore-forming strains that survive gastrointestinal transit effectively may be readily detected in fecal samples, whereas transient colonizers of mucosal surfaces may be substantially underestimated. 

This focused narrative review examines the limitations of fecal sampling in probiotic research and discusses methodological strategies to improve the reliability of this approach, thereby enhancing our understanding of how probiotics influence the gut microbiome.

## Review

Evidence against the exclusive use of fecal specimens

A growing body of gastroenterological research indicates that stool samples do not reliably reflect the intestinal mucosal microbiome. Nevertheless, fecal samples may capture important functional signals, including short-chain fatty acid concentrations and immune-modulating metabolites, even when their microbial composition differs from that of the mucosa. In a culture-based study of Beagle dogs, researchers observed qualitative and quantitative differences between jejunal and corresponding fecal samples [2]. Although this older culture-based study has methodological limitations compared with contemporary sequencing approaches, its findings remain conceptually relevant. Similarly, in a rat trinitrobenzene sulfonic acid (TNBS) colitis model, treatment with the probiotic VSL#3^®^ (VSL Pharmaceuticals, Herndon, USA) further reduced luminal microbial diversity, already diminished by colitis, but had no measurable effect on the mucosa-associated microbiome. These findings illustrate that fecal observations may not accurately reflect events occurring at the mucosal surface [3]. The probiotic formulation marketed as VSL#3 before 2016 is now formally designated the De Simone Formulation. The product currently marketed as VSL#3 is not equivalent to the De Simone Formulation because its composition was altered after the 2016 formulation change [4]. 

Human studies provide additional evidence of differences between fecal and mucosal microbial communities. Analysis of saliva, mucosal, and fecal samples from 21 healthy adults showed that individual intestinal regions harbor distinct bacterial communities and that fecal microbiota do not accurately reflect the mucosal microbiome [5,6]. Similarly, a sequencing-based study comparing stool and sigmoid brush samples from 40 adults found that relying on fecal samples alone may underestimate microbial richness and diversity [7]. In studies of inflammatory bowel disease, mucosal microbiota were more effective than fecal microbiota at distinguishing disease subtypes [8,9]. Although these observations are compelling, they should be interpreted cautiously given the disease-specific populations and the relatively small cohorts. Less invasive intermediate approaches, such as rectal swabs or sigmoidoscopy-guided brush sampling, may offer a practical compromise between fecal sampling and full endoscopic mucosal sampling in future probiotic studies. 

A culture-based study of individuals receiving *Lactobacillus plantarum* 299v found an increase in Clostridia in fecal samples but not in rectal or ascending-colon mucosal samples, further demonstrating the site-specific nature of probiotic-associated microbial changes [10]. Finally, a large, carefully designed study showed that resistance to mucosal colonization by empirically administered probiotics was associated with distinct host and microbiome characteristics. In both the murine and human intestinal tracts, the mucosal microbiome correlated only partially with findings from stool samples [11].

Strategies to improve fecal sampling

Although stool specimens may not fully capture microorganisms associated with intestinal tissue, relatively few animal and human studies have directly examined probiotic effects on the microbiota in situ [12]. Sampling multiple intestinal sites requires invasive endoscopic procedures that are technically challenging, burdensome for participants, and costly. Consequently, fecal sampling remains the predominant approach in clinical microbiome research. The strategies discussed below address two conceptually distinct issues. The first group, including multi-day collection, optimized DNA extraction, and quantitative profiling, aims to improve the accuracy and representativeness of information obtained from fecal specimens. The second seeks to bypass fecal sampling by directly sampling defined intestinal regions with ingestible devices. Each strategy addresses different limitations and entails specific practical trade-offs.

Sample Number and Collection Methods

The accuracy and reproducibility of gut microbiome studies depend not only on sequencing and bioinformatic methods but also on fecal specimen collection, processing, and storage. Despite their importance, these pre-analytical factors remain insufficiently standardized. 

An important consideration is whether a single fecal specimen adequately represents an individual's overall gut microbial community. Because intestinal microbial composition may fluctuate day to day, relying on a single sample may miss normal intra-individual variability. Studies using repeated fecal collections have demonstrated measurable temporal variation in microbial composition, suggesting that repeated sampling may provide a more representative assessment, particularly when subtle changes related to diet, disease, or an intervention are under investigation.

The optimal number of specimens should therefore depend on the study objective. In large clinical or epidemiological studies, repeated sampling on consecutive days may improve reliability, though it also increases participant burden and study costs. A study comparing one-day and five-day fecal collection protocols for DNA-based gut microbiota analysis found significantly greater alpha diversity in five-day samples than in single-day samples [12]. However, variability in the abundance of individual bacterial genera was also more pronounced. Repeated sampling may therefore be particularly important when the primary outcome focuses on specific microbial taxa rather than overall diversity. Multi-sample collection strategies are also used in other areas of gastroenterology, including colorectal cancer screening [13]. The method used to obtain a fecal specimen is another important consideration. Spot sampling from a single region of the stool may yield variable results because microorganisms are not necessarily distributed uniformly throughout the specimen. Studies have shown differences in microbial detection across regions of the same stool. Homogenizing the entire specimen before subsampling can reduce this sampling bias and produce aliquots that are more representative of the complete specimen [14]. Collection timing and storage conditions are also critical. Microbial composition and metabolite concentrations may change after defecation, especially with prolonged exposure to room temperature and oxygen [15]. Whenever feasible, participants should collect the first complete bowel movement of the day using a standardized procedure and freeze the specimen immediately after collection. Consistency in collection time, storage temperature, container type, and processing procedures is especially important when comparing samples longitudinally or between study groups [16]. 

Overall, microbiome studies should adopt standardized fecal-sampling protocols that specify the number and timing of collections, whole-stool versus spot sampling, homogenization, and storage conditions. Careful attention to these seemingly simple methodological factors can substantially improve the reliability, reproducibility, and interpretability of gut microbiome research [14].

DNA Extraction Methods

Accurate characterization of the human gut microbiome depends not only on appropriate fecal sample collection and storage but also on the DNA extraction method used before sequencing. Because fecal specimens contain highly diverse microbial communities, extraction methods should efficiently lyse microorganisms with different cell-wall structures while yielding sufficient DNA quantity and quality for downstream molecular analyses. Importantly, the extraction procedure itself may introduce substantial technical bias, thereby influencing the apparent composition and diversity of the gut microbiome. A particularly important consideration is including a mechanical disruption step, such as bead beating, during DNA extraction. Gram-positive bacteria and other microorganisms with relatively resistant cell walls may be difficult to lyse using chemical or enzymatic procedures alone, leading to underrepresentation of their DNA in the final extract. Mechanical disruption with beads can improve cell lysis and increase DNA recovery from these organisms, potentially providing a more comprehensive representation of the microbial community. Lim et al. compared nine DNA extraction protocols, including three commercial kits and combinations of manual or automated procedures, with or without an additional bead-beating step. Although all methods produced DNA of sufficient concentration and quality for sequencing, the resulting microbiome profiles varied by extraction procedure. Most importantly, bead beating was associated with higher measured microbial diversity and substantial differences in observed gut microbiome composition. These findings show that DNA extraction is not merely a preliminary technical step but a key determinant of the microbiological information obtained from a fecal specimen [17]. Other studies have reached similar conclusions. Wu et al. reviewed the entire process from fecal collection through DNA extraction and emphasized the lack of a universally accepted standardized protocol for microbiome studies. Differences in sample handling, storage conditions, DNA stabilization, aliquoting, and extraction procedures can all contribute to technical variability across studies. Standardization is therefore particularly important when samples are collected at multiple centers or at different time points within clinical studies [18]. 

In studies examining the effects of dietary interventions, probiotics, prebiotics, medications, or disease on the gut microbiome, the same DNA extraction protocol should be used for all specimens. Ideally, study designs should also include technical replicates, negative extraction controls, and, where appropriate, mock microbial communities. Consistent sample processing and detailed documentation of extraction procedures can reduce methodological bias and improve reproducibility [19]. DNA extraction should therefore be regarded as an integral component of fecal microbiome study design rather than merely a laboratory processing step. In particular, incorporating effective mechanical lysis, such as bead beating, may improve recovery of difficult-to-lyse organisms and yield a more representative assessment of gut microbial diversity and composition.

Quantitative Microbiome Profiling

Current sequencing-based analyses of the fecal microbiota typically quantify microbial taxa and metabolic pathways as proportions of the total sequencing library. These relative-abundance measurements have been extremely valuable for identifying associations between the gut microbiome and health or disease. However, they characterize microbial community composition without directly indicating the total number of microorganisms in a fecal specimen. Consequently, an apparent increase in the relative abundance of a bacterial taxon may reflect a reduction in other taxa rather than a genuine increase in the absolute number of that organism. This limitation is particularly important when microbial load varies substantially among individuals or between samples from the same individual at different time points. In these cases, relative microbiome profiling may obscure biologically meaningful changes and complicate efforts to link microbiota characteristics to host physiological or metabolic parameters. For example, a bacterial species may remain stable or even decrease in absolute abundance while appearing to increase proportionally because the overall microbial load has declined [19]. Vandeputte et al. highlighted this issue by developing a quantitative microbiome-profiling approach that combined sequencing with flow cytometry-based direct enumeration of microbial cells. They demonstrated substantial variation in fecal microbial load among healthy individuals and showed that microbial abundance could explain key aspects of microbiome variation and its relationship to host phenotype. Importantly, some relationships among bacterial groups identified by conventional relative-abundance analyses appeared to be compositional artifacts rather than true biological interactions. In patients with Crohn's disease, microbial load itself was identified as an important determinant of observed microbiota alterations [20]. Several approaches can yield absolute rather than exclusively relative measurements, including flow cytometry, quantitative PCR (qPCR), digital PCR, and defined microbial or DNA spike-in standards. Each approach has advantages and limitations, and the optimal method depends on the research question, sample type, microbial biomass, and available laboratory resources [21].

A practical approach for fecal microbiome studies is to quantify total bacterial load using 16S ribosomal RNA (rRNA) gene qPCR and to combine this measurement with sequencing-derived relative abundances. This enables conversion of relative abundance data into estimates of absolute abundance. Quantitative profiling can thereby distinguish a genuine increase or decrease in a bacterial taxon from an apparent change resulting from shifts in the overall microbial community [22]. Accordingly, when investigating how disease, diet, probiotics, prebiotics, medications, or other interventions affect the gut microbiome, reporting relative abundance alone may provide an incomplete picture. Whenever feasible, fecal microbiome studies should estimate total microbial load and ideally report both relative and absolute abundances. This approach can improve the biological interpretation of microbiome data and strengthen conclusions about host-microbiota interactions [20-22].

Novel Non-invasive Techniques for Direct Microbiome Sampling

Current approaches to studying the gut microbiota rely predominantly on fecal specimens, intestinal aspirates, mucosal biopsies, or endoscopic procedures. Fecal sampling is attractive because it is simple, inexpensive, non-invasive, and suitable for repeated collection. However, it provides only an indirect representation of the intestinal microbiome. A stool specimen contains material from multiple regions of the gastrointestinal tract and therefore cannot reliably capture the spatial organization of microbial communities along the intestinal axis. Furthermore, luminal and mucosa-associated microbiota may occupy distinct ecological niches with different bacterial compositions and functions. Reliance on fecal specimens may therefore obscure important site-specific microbial characteristics and host-microbe interactions. Endoscopic biopsy offers greater anatomical precision and enables direct investigation of mucosa-associated microorganisms. However, it is invasive, often requires bowel preparation and sedation, samples only a limited area of the mucosa, and is unsuitable for frequent longitudinal sampling, particularly in healthy individuals. Biopsy specimens may also contain substantial amounts of host DNA, which can complicate microbiome analyses [23,24]. These limitations have spurred the development of novel technologies to obtain microbiological samples from defined gastrointestinal locations without conventional endoscopy [25,26]. 

One promising approach is an electronics-free ingestible capsule designed for targeted microbiome sampling in the proximal colon. Nejati et al. developed a 3D-printed smart capsule containing a superabsorbent hydrogel and a flexible polydimethylsiloxane disk. A double-layer, pH-sensitive enteric coating protects the sampling aperture and prevents luminal contents from reaching the sampling chamber until the capsule reaches the proximal colon. When the appropriate gastrointestinal pH is encountered, the coating dissolves, allowing sampling. The device then closes, preserving the collected material. The optimal coating formulation was established through dissolution experiments conducted under simulated gastrointestinal conditions. Sampling performance and microbial preservation were then evaluated using realistic in vitro gastrointestinal models containing mixed bacterial cultures and in vivo porcine experiments. Importantly, 16S rRNA and whole-genome sequencing analyses showed that bacterial populations retrieved by the capsule closely mirrored those in the targeted proximal-colon region [26]. Such technologies may represent an important advance toward more precise characterization of the intestinal microbiome. Site-specific sampling could improve understanding of microbial biogeography, diet-microbiome interactions, probiotic and prebiotic effects, and regional microbial alterations linked to gastrointestinal diseases. Nevertheless, further validation in humans is required before these devices can be routinely used in clinical or research practice [27]. Future development should focus on improving sampling accuracy, preserving anaerobic microorganisms and microbial metabolites, facilitating device retrieval, ensuring patient safety, and enabling sampling from multiple gastrointestinal sites during a single transit.

## Conclusions

Fecal sampling remains the most widely used method for assessing the effects of probiotic agents on the gut microbiota in clinical research. Although this approach offers substantial practical advantages, its major limitation is that it incompletely represents the mucosa-adherent microbiome. Nevertheless, fecal specimens remain valuable for detecting broad changes in the microbial community, assessing functional readouts, and supporting longitudinal studies. The methodological strategies discussed in this review, including standardized and repeated sample collection, optimized DNA extraction, quantitative microbiome profiling, and novel direct-sampling devices, could substantially improve the reliability and depth of future probiotic microbiome studies. 

An important direction for future research would be systematic studies that directly compare fecal, mucosal-biopsy, and capsule-derived microbiome profiles in the same participants. Such studies could help define the circumstances under which each sampling approach provides the most informative representation of probiotic effects on the gut microbiome. International organizations such as the International Probiotics Association (IPA) and the International Scientific Association for Probiotics and Prebiotics (ISAPP) may wish to consider how such evidence, once available, could inform future methodological guidance
